# Health Literacy and Personality Traits in Two Types of Family Structure—A Cross-Sectional Study in China

**DOI:** 10.3389/fpsyg.2022.835909

**Published:** 2022-04-25

**Authors:** Jianrong Mai, Wu Yibo, Zhou Ling, Lin Lina, Sun Xinying

**Affiliations:** ^1^School of Nursing, Guangzhou Xinhua University, Guangzhou, China; ^2^School of Public Health, Peking University, Beijing, China

**Keywords:** health literacy, personality traits, family structure, health outcomes, health promotion

## Abstract

**Objective:**

The level of health literacy is one of the important factors affecting health outcomes. Family is an important place to shape personality traits, and people with different personalities will adopt different lifestyles, which will lead to variations in health outcomes. Therefore, this article aims to explore the relationship between health literacy and personality and its influencing factors in different family structures.

**Methods:**

This was a cross-sectional study with 1,406 individuals. A questionnaire was utilized to measure health literacy, personality and demographic variables, including family structure. Canonical correlation analysis (CCA) and hierarchical multiple regression analysis were used to examine the relation between health literacy and personality traits between two types of family structure.

**Results:**

CCA showed that the canonical correlation coefficients were 0.309 (*p* < 0.001) and 0.347 (*p* < 0.001), in two-parent family and single-parent family, respectively. The openness of personality traits exhibited the highest correlation with health literacy. Compared with the remaining personality traits, openness yielded the strongest effect (β = 0.485 and β = 0.830) in two types of family structure, respectively. Education and monthly income were significantly associated with health literacy.

**Conclusion:**

Our results support the relation between health literacy and personality traits in two types of family structure.

## Introduction

People are more sensitive and pay more attention to health information after COVID-19. The interpretation of health information is an important part of health literacy, which includes three dimensions: healthcare, disease prevention, and health promotion (Duong et al., 2019). Health literacy is a necessary skill and resource for people to seek, understand, evaluate, communicate, and use health information and services throughout their lives to promote health (Sorensen et al., 2012). Health literacy can be an independent factor influencing health outcomes. Those with a low level of health literacy are related to poor health outcomes such as insufficient use of healthcare services, high hospitalization rates, and high mortality rates according to some studies (DeWalt et al., 2004; Baker et al., 2007; Huang et al., 2017; Iwasa and Yoshida, 2020). In addition, it is difficult for people with low health literacy to update their health knowledge or obtain the best medical advice from health experts to change their health-related lifestyle. Individuals who have higher awareness of health literacy are more willing to establish health behavior and lifestyle. Some studies reported that socioeconomic status (SES) had closely links to health literacy (Adler and Newman, 2002; Bonaccorsi et al., 2019). SES may become a predictor of health literacy (Iwasa and Yoshida, 2020).

In 2020, the health literacy level of Chinese residents reached 23.15%, an increase of 3.95 percentage points from 2019, and the growth rate is the largest in history (National Health Commission of the People’s Republic of China, 2021). People have taken the initiative to assume social responsibilities, learn the knowledge and skills of epidemic prevention and control during the COVID-19 pandemic. Some methods and information can be transmitted to those who have lower health literacy, and thus they are encouraged to identify health information, master health promotion skills, and develop health behaviors. Health caregivers should pay attention not only to the transmission of health knowledge, but also to the training of health skills (e.g., strengthening the awareness of physical examination, quitting smoking, maintaining a good mental state, and avoiding natural disasters) in health promotion. Some studies on health literacy have revealed that health outcomes are associated with factors such as personality traits and family structure (Huang et al., 2017; van Scheppingen et al., 2019; Iwasa and Yoshida, 2020; Sentell et al., 2020; Soutter et al., 2020; Harsch et al., 2021).

A previous study reported the relationship between personality traits and health literacy among older adults living in Japan, and pointed out some factors of personality traits (e.g., extraversion, openness, and conscientiousness) may influence the health literacy (Iwasa and Yoshida, 2020). The Big Five Personality traits include five categories: extraversion (being positive and confident), agreeableness (being trusting and generous), conscientiousness (being rational and self-disciplined), neuroticism (being anxious and depressive) and openness (being curious and audacious) (Norman, 1963; McCrae and John, 1992; Getzmann et al., 2021). Compared with introverts, extroverts respond more strongly to positive emotions and are more likely to experience delightful mood (Soutter et al., 2020). Those with a high level of agreeableness can make good use of problem-focused and positive emotion-focused strategies, such as seeking social support and searching comprehensive information online and reducing negative emotion (Connor-Smith and Flachsbart, 2007; Carver and Connor-Smith, 2010; Getzmann et al., 2021). Additionally, people high in conscientiousness are more likely to search health information of recent health concerns for a family member (Bogg and Vo, 2014). Neuroticism is related to negative emotions, such as anxiety and depression. Individuals with a high level of neuroticism are more likely to rely on negative emotion-focused coping strategies that can easily lead to mood changes, eating disorders and drug abuse (Aschwanden et al., 2020; Herke et al., 2020; Getzmann et al., 2021). People with a high level of openness are full of curiosity, imagination and creativity and are more likely to take action emotionally. They are interested in new ideas and new experiences, so that they are more willing to search and collect health information (Bogg and Vo, 2014).

Family structure is an important factor that influences children’s health literacy, personality traits, economic, and social resources (Bloom and Dawson, 1991; Bogg and Roberts, 2004; Chae, 2016; Berger-Sieczkowski et al., 2019; Lastrucci et al., 2019; Segerstrom, 2020). In our study, we focused on two types of family structure: two-parent family and single-parent family. The two-parent family is composed of parents and unmarried children for the entirety of childhood. A single-parent family is held by one biological parent and the other is divorced, remarried, or widowed. People who grow up in two-parent family may have fewer internalizing (e.g., fearfulness, depression, and social withdrawal problems) and externalizing (e.g., aggression, hyperactivity, and oppositionality behaviors) problems than those from single-parent family (Campbell, 1995; Amato and DeBoer, 2001; Christopher et al., 2017). Low scores of conscientiousness and agreeableness have been shown to have associations with externalizing and internalizing behavior problems (Roberts et al., 2009; Laursen et al., 2010; Kim and Kochanska, 2019). Adolescents can be more extroverted and agreeable in a warm, understanding, and supportive family (Bloom and Dawson, 1991; Yong-bao and Jing, 2019).

Different than Iwasa’s study that focused on the relationship between SES and health literacy among older adults living in Japan, the present study looks into the relationship between health literacy and personality traits. Factors such as extraversion, agreeableness, conscientiousness, neuroticism and openness may be predictors of health outcomes and clarify the mechanism between health literacy and personality traits in Chinese population. As such, this study aims to explore the relationship between health literacy and personality traits among two types of family structure, and provides a basis for studying the mechanism of personality traits and health literacy.

## Materials and Methods

### Participants and Procedure

This is a subproject of the Survey on Health Index Among Chinese Families [SHIACF] (2021) that aims at studying the health status among Chinese families. A cross-sectional study in mainland China was conducted between July and September 2021. According to the data of “Seventh National Census in 2020,” 120 cities in 23 provinces, 5 autonomous regions, and 4 municipalities directly under the Central Government were randomly selected. Teams were recruited to ensure the implementation of the investigation in each city. The study was conducted between July and September 2021 using an online questionnaire. A total of 1,406 individuals were selected to participate in this study using multistage random sampling method. Participants then outlined their family structure (e.g., single-parent family or two-parent family). Demographic variables such as gender, age, educational level, residential area, and monthly income were inquired and used as factors associated with health literacy in hierarchical multiple regression analysis. The inclusion criteria for participants were age ≥ 12, living in China, volunteering to participate in the research and completing the consent form. The exclusion criteria were participants with severe mental illness and unwillingness to cooperate. All participants provided consent form and this study was approved by the Ethics Committee of Jinan University, Guangzhou, China.

Among 1,406 respondents, 610 (43.4%) were male and 796 (56.6%) were female; 994 (70.7%) were from two-parent family and 29.3% (412) were from single-parent family. A total of 156 (11.1%) of participants were under 18 years old, 1,173 (83.4%) were between 19 and 59, and 77 (5.5%) were over 60 (Table 1).

**TABLE 1:** Sample characteristics (*N* = 1,406).

Characteristics of participants	Variables	Number (%)
Gender	Male	610 (43.4)
	Female	796 (56.6)
Age group	<19	156 (11.1)
	19–59	1,173(83.4)
	>59	77 (5.5)
Education	Illiteracy	33 (2.3)
	Primary school	89 (6.3)
	Middle school	193 (13.7)
	High school	278 (19.9)
	Junior college	163 (11.6)
	Undergraduate or higher	650 (46.2)
Family structure	Two-parent family	994 (70.7)
	Single-parent family	412 (29.3)
Residential area	Urban area	1,016(72.3)
	Rural area	390 (27.7)
Monthly income	<1,501	152 (10.7)
	1,501–4,500	573 (40.8)
	4,501–9,000	465 (33.1)
	>9,000	216 (15.4)

### Measures

#### Health Literacy

Health literacy was measured by a 12-item short-form health literacy questionnaire (HLS-SF12), which was developed and based on the 47-item European health literacy questionnaire (HLS-EU-Q47) (Duong et al., 2019). The HLS-SF12 was a self-report questionnaire comprising three domains: healthcare, disease prevention, and health promotion. The difficulty of HLS-SF12 was rated on a four-point Likert scale (1, very difficult; 2, difficult; 3, easy; 4, very easy). The cumulative score of each domain comprises the total score of HLS-SF12. Higher scores indicated a higher level of health literacy. Cronbach’s alpha was 0.937 for the present study. The HLS-SF12 was proved to have adequate reliability and validity.

#### Personality Traits

The Big Five Inventory-10 (BFI-10) offered a sufficient evaluation of personality traits including extraversion, agreeableness, conscientiousness, neuroticism and openness. Respondents rated each item on a five-point Likert scale (1, disagree strongly; 2, disagree; 3, neither disagree nor agree; 4, agree; and 5, agree strongly). Cronbach’s alpha was 0.486 for the present study. The BFI-10 retained significant levels of validity and reliability (Rammstedt and John, 2007).

### Statistical Analysis

Data were analyzed using IBM SPSS version 25. First, we computed the descriptive statistics and measured the health literacy score using ANOVA between demographic variables. Student’s *t*-test was performed to identify the personality score in two types of family structure. Next, to assess the association between personality traits and health literacy, Pearson correlation and canonical correlation analysis (CAA) were computed in two-parent family and single-parent family, respectively. Extraversion, agreeableness, conscientiousness, neuroticism, and openness were selected as dependent variable set owing to describe the personality traits. Healthcare (HC), disease prevention (DP), and health promotion (HP) were selected as independent variable set owing to health literacy. Furthermore, hierarchical multiple regression analysis was used to test health literacy as a dependent variable and residential area, monthly income, gender, education, family structure, and personality traits as predictors. Significance was set at *p* < 0.05 (two-sided).

## Results

### Differences of Health Literacy by Demographic Variables

The demographic characteristics of the study are presented in Table 2. There were significant differences between health literacy and variables of family structure, residential area, monthly income, and education (*p* < 0.05).

**TABLE 2:** Comparison of health literacy between demographic characteristics (*N* = 1,406).

Demographic characteristics	(Mean ± SD)	*F*	*p*
Family structure		2.962	0.003
Two-parent family	37.23 ± 5.41		
Single-parent family	36.25 ± 6.26		
Residential area		5.319	<0.001
Urban area	37.44 ± 5.40		
Rural area	35.65 ± 6.21		
Monthly income (RMB)		11.615	<0.001
≤1,500	34.80 ± 0.55		
1,500–4,500	36.65 ± 0.23		
4,501–9,000	37.58 ± 0.28		
≥9,001	37.82 ± 0.36		
Education		19.363	<0.001
Illiteracy	31.58 ± 1.37		
Primary school	33.07 ± 0.61		
Middle school	36.03 ± 0.37		
High school	37.33 ± 0.35		
Junior college	37.31 ± 0.38		
Undergraduate or higher	37.75 ± 0.22		
Gender		0.738	0.461
Male	37.07 ± 6.01		
Female	36.84 ± 5.43		

### Personality Traits in Two Types of Family Structure

Table 3 shows the comparison of personality scores between two-parent family and single-parent family. The mean ± SD of A (agreeableness) was significantly different between the two groups (*p* = 0.003). This result indicated that individuals in the two-parent family had a higher level of agreeableness than those in the single-parent family.

**TABLE 3:** Comparison of personality traits between two-parent family and single-parent family.

	E	A	C	N	O
Two-parent family	6.32 ± 1.69	7.10 ± 1.48	6.82 ± 1.68	6.28 ± 1.53	6.44 ± 1.57
Single-parent family	6.47 ± 1.66	6.84 ± 1.49	6.68 ± 1.55	6.19 ± 1.47	6.54 ± 1.52
*t*	1.482	3.013	1.493	0.944	1.131
*p*	0.139	0.003*	0.136	0.346	0.258

*p < 0.05.

*E, extraversion; A, agreeableness; C, conscientiousness; N, neuroticism; O, openness.*

### Bivariate Correlation Between Health Literacy and Personality Traits

Bivariate correlations among variables of health literacy and personality traits are shown in Table 4. All variables related to personality traits exhibited positively significant correlations with health literacy such as HC, DP, and HP (*p* < 0.05).

**TABLE 4:** Correlation matrix for the variables of health literacy and personality traits.

Variables	E	A	C	N	O	HC	DP	HP
E	1.000	−	−	−	−	−	−	−
A	–0.015	1.000	−	−	−	−	−	−
C	0.139*	0.262*	1.000	−	−	−	−	−
N	0.162*	0.239*	0.123*	1.000	−	−	−	−
O	0.199*	0.128*	0.075*	0.090*	1.000	−	−	−
HC	0.082*	0.128*	0.061*	0.116*	0.175*	1.000	−	−
DP	0.126*	0.151*	0.106*	0.118*	0.218*	0.792*	1.000	−
HP	0.117*	0.182*	0.146*	0.097*	0.235*	0.709*	0.781*	1.000

*p < 0.05.

*E, extraversion; A, agreeableness; C, conscientiousness; N, neuroticism; O, openness; HC, healthcare; DP, disease prevention; HP, health promotion.*

### Results of Canonical Correlation Analysis

Although the bivariate correlation showed a positively significant correlation between the variables of health literacy and personality traits, it was difficult to interpret the relation between two sets of variables. Thus, CCA may offer an efficient way to assess this association between two sets of variables.

#### Results of Canonical Correlation

The present study defined the variables of personality traits as the independent variables and health literacy as the dependent variable. Table 5 shows that the first canonical correlation was 0.309 in the two-parent family and 0.347 in the single-parent family. The first canonical correlation interpreted the highest possible correlation between all linear combinations for two sets of variables. The second canonical correlations were 0.131 and 0.071, respectively. Although the canonical correlation of 0.131 was significant, the proportion of variance explained was less than the first canonical correlation. Therefore, our study considered retaining the first canonical correlation coefficient.

**TABLE 5:** Results of canonical correlation in two types of family structure.

Pair	Canonical correlation	Eigenvalue	Wilks statistic	*F*	*p*	PVE (set 1/set 2)
**Two-parent family**						
1	0.309	0.105	0.887	8.037	0.000	0.310/0.729
2	0.131	0.017	0.981	2.404	0.014	0.185/0.266
3	0.046	0.002	0.998	0.701	0.552	0.200/0.072
**Single-parent family**						
1	0.347	0.137	0.873	3.766	0.000	0.330/0.858
2	0.071	0.005	0.992	0.422	0.908	0.131/0.125
3	0.057	0.003	0.997	0.440	0.724	0.164/0.110

*PVE, proportion of variance explained.*

The standardized canonical coefficients of E, A, C, N, and O as the variables of personality traits in the parent family were −0.150, −0.359, −0.410, −0.111, and −0.641, respectively, for the first canonical variate (VT_1_). HC, DP, and HP were the variables of health literacy, and the standardized canonical coefficients were 0.192, −0.195, and −0.973, respectively, for the first canonical variate (WT_1_). The coefficients of O (−0.641) and HP (−0.973) indicated that they contributed the most to personality traits and health literacy in the two-parent family, respectively.

In the single-parent family, the standardized canonical coefficients of E, A, C, N, and O were −0.337, −0.424, −0.050, −0.015, and −0.659, respectively, for the first canonical variate (VS_1_). The first canonical variate (WS_1_) of health literacy, such as HC, DP, and HP were 0.273, −0.780, and −0.497, respectively. It demonstrated that O (−0.659) and DP (−0.780) contributed the strongest to personality traits and health literacy (Table 6).

**TABLE 6:** Standardized canonical coefficients for personality traits and health literacy in two types of family structure.

	Personality traits variable set						Health literacy variable set		
	E	A	C	N	O		HC	DP	HP
**Two-parent family**									
VT_1_	–0.150	–0.359	–0.410	–0.111	–0.641	WT_1_	0.192	–0.195	–0.973
VT_2_	–0.205	0.025	0.642	–0.813	–0.065	WT_2_	–0.915	–0.717	1.146
VT_3_	–0.885	0.403	–0.157	0.181	0.112	WT_3_	1.334	–1.711	0.531
**Single-parent family**									
VS_1_	–0.337	–0.424	–0.050	–0.015	–0.659	WS_1_	0.273	–0.780	–0.497
VS_2_	–0.762	–0.543	0.004	0.330	0.788	WS_2_	1.331	0.339	–1.429
VS_3_	–0.118	0.594	–0.964	0.061	–0.010	WS_3_	1.188	–1.772	0.894

*E, extraversion; A, agreeableness; C, conscientiousness; N, neuroticism; O, openness; HC, healthcare; DP, disease prevention; HP, health promotion; VT_1_, VT_2_, and VT_3_, personality traits variables for two-parent family; WT_1_, WT_2_, and WT_3_, health literacy variables for two-parent family; VS_1_, VS_2_, and VS_3_, personality traits variables for single-parent family; WS_1_, WS_2_, and WS_3_, health literacy variables for single-parent family.*

The optimal linear equations were:

VT_1_ = −0.150*E-0.359*A-0.410*C-0.111*N-0.641*O

WT_1_ = 0.192*HC-0.195*DP-0.973*HP

VS_1_ = −0.337*E-0.424*A-0.050*C-0.015*N-0.659*O

WS_1_ = 0.273*HC-0.780*DP-0.497*HP

#### Results of Canonical Loading

According to canonical loading results in the two-parent family, O (−0.745) had a stronger effect than E (−0.314) and N (−0.337) to form the first pair variable for personality traits (VT_1_). Additionally, HP (−0.992) for health literacy had greater effect than HC (−0.621) and DP (−0.792) to form the first pair (WT_1_). Similarly, canonical loading of O (−0.839) played a role in personality traits compared to other factors in the single-parent family (VS_1_). However, canonical loading for health literacy indicated that DP (−0.957) and HP (−0.915) had a greater effect than HC (−0.739) in forming the first pair (ST_1_) (Table 7).

**TABLE 7:** Canonical loadings for personality traits and health literacy in two types of family structure.

	Personality traits variable set						Health literacy variable set		
	E	A	C	N	O		HC	DP	HP
**Two-parent family**									
VT_1_	–0.314	–0.558	–0.581	–0.337	–0.745	WT_1_	–0.621	–0.792	–0.992
VT_2_	–0.263	–0.006	0.501	–0.759	–0.124	WT_2_	–0.695	–0.550	–0.027
VT_3_	–0.872	0.448	–0.151	0.112	0.032	WT_3_	0.362	–0.264	0.124
**Single-parent family**									
VS_1_	–0.568	–0.561	–0.282	–0.227	–0.839	WS_1_	–0.739	–0.957	–0.915
VS_2_	–0.487	–0.359	–0.158	0.150	0.488	WS_2_	0.523	0.277	–0.147
VS_3_	–0.220	0.323	–0.803	0.119	–0.037	WS_3_	0.425	–0.090	0.375

*E, extraversion; A, agreeableness; C, conscientiousness; N, neuroticism; O, openness; HC, healthcare; DP, disease prevention; HP, health promotion; VT_1_, VT_2_, and VT_3_, personality traits variables for two-parent family; WT_1_, WT_2_, and WT_3_, health literacy variables for two-parent family; VS_1_, VS_2_, and VS_3_, personality traits variables for single-parent family; WS_1_, WS_2_, and WS_3_, health literacy variables for single-parent family.*

**FIGURE 1:**
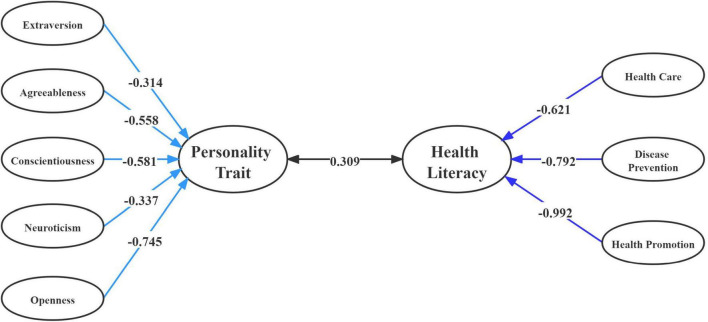
Correlations between variables set of personality traits and variables set of health literacy and among their canonical variables in two-parent family.

**FIGURE 2:**
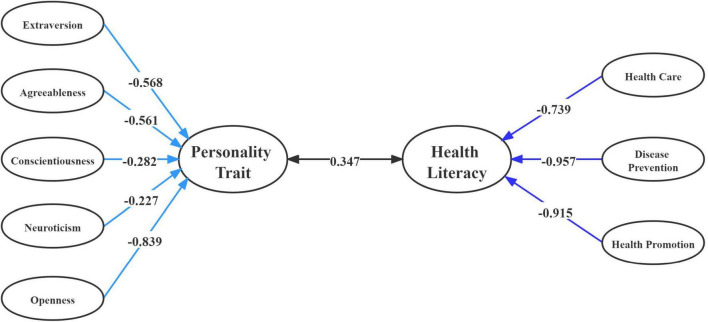
Correlations between variables set of personality traits and variables set of health literacy and among their canonical variables in single-parent family.

Thus, a related structural illustration of the first canonical correlation on personality traits and health literacy in two-type family structures is shown in Figures 1, 2.

### Determinants of Health Literacy in Two Types of Family Structure

A hierarchical multiple regression analysis, which was based on the results of CAA, was used to examine the factors associated with health literacy. The current study selected residential area, monthly income, gender, education, and personality traits as the independent variables. Regression coefficients were performed in Table 8.

**TABLE 8:** Factors associated with health literacy in two types of family structure.

	Two-parent family				Single-parent family			
	Model 1		Model 2		Model 3		Model 4	
	β	*P*	β	*P*	β	*P*	β	*P*
Constant	31.595	< 0.001	21.227	< 0.001	31.626	< 0.001	20.391	< 0.001
Residential area (ref. = urban area)	–0.522	0.237	–0.593	0.167	–0.418	0.612	–0.586	0.466
Monthly income (ref. = <1,500)	−	−	−	−	−	−	−	−
Monthly income (1,500–4,500)	0.197	0.762	0.203	0.747	1.910	0.033	1.523	0.080
Monthly income (4,501–9,000)	0.676	0.313	0.550	0.395	2.504	0.012	2.141	0.027
Monthly income (≥9,001)	0.752	0.316	0.711	0.326	2.121	0.088	1.590	0.188
Gender (ref. = female)	–0.401	0.311	–0.400	0.300	0.323	0.640	0.136	0.840
Education (ref. = illiteracy)	−	−	−	−	−	−	−	−
Education (primary school)	2.432	0.110	2.331	0.114	0.349	0.849	0.112	0.950
Education (middle school)	4.829	0.001	4.678	0.001	3.631	0.033	2.650	0.114
Education (high school)	6.995	< 0.001	6.818	< 0.001	1.961	0.235	1.587	0.329
Education (junior college)	6.034	< 0.001	5.886	< 0.001	3.810	0.030	2.997	0.080
Education (undergraduate or higher)	6.752	< 0.001	6.563	< 0.001	3.687	0.020	2.818	0.072
Extraversion	−	−	0.222	0.025	−	−	0.270	0.162
Agreeableness	−	−	0.288	0.014	−	−	0.529	0.015
Conscientiousness	−	−	0.325	0.002	−	−	0.080	0.702
Neuroticism	−	−	0.259	0.021	−	−	0.083	0.696
Openness	−	−	0.485	< 0.001	−	−	0.830	< 0.001
*R* ^2^	0.094	−	0.160	−	0.064	−	0.134	−

First, all factors, except personality traits, were selected into model 1 (two-parent family without personality traits factors) and model 3 (single-parent family without personality traits factors). Education proved to be statistically significant in both regression models. As the educational level increased, health literacy increased. Additionally, monthly income was another factor that influenced health literacy in the single-parent family.

Next, all factors including personality traits selected through CCA, were included in model 2 (two-parent family with personality trait factors) and model 4 (single-parent family with personality trait factors). Education remained a predictor in the regression model of the two-parent family. All five personality trait factors were statistically significant in model 2, which that they were predictors of health literacy in the two-parent family. The coefficient of determination, *R*^2^, varied from 0.094 to 0.160 in model 1 and model 2. Monthly income (for 4,501–9,000), agreeableness and openness of personality traits were significant in model 4. However, education was no longer significant in this regression. The *R*^2^ was 0.134 in model 4 (Table 8).

## Discussion

To our best knowledge, the present study is the first to focus on the relationship between health literacy and personality traits in China. We found that education, extraversion, agreeableness, conscientiousness, neuroticism, and openness were significantly related to health literacy in two-parent family. However, monthly income, agreeableness, and openness were influencing factors of health literacy in single-parent family.

The average score of health literacy was 36.94 ± 5.69 in our study. Comparing the results with Duong, people in mainland China had a higher level of health literacy than the other six countries or areas in Asia (*p* < 0.05) (Duong et al., 2019). Our study identified that health literacy was associated with education. Health literacy is considered to be able to collect, read, comprehend, and utilize health resources (Zarcadoolas et al., 2005; Manganello, 2007). Education for health was considered to be a health promotion action for the general population (Nutbeam, 2000). Those with a higher level of education have been confirmed to have higher cognitive functions, which are an effective skills to collect health information (Iwasa and Yoshida, 2020). Monthly income is another factor that influenced the health literacy. Individuals with a reasonable economic status are more likely to make good use of healthcare resources and to collect health information in the right way (Nutbeam, 2000; Wharf Higgins et al., 2009; Sorensen et al., 2012; Rask et al., 2014; Martin and Chen, 2015). In addition, high monthly income indicates that people can widely select health resources, health insurance, and healthcare systems (Ishikawa et al., 2018).

This study found that individuals with higher agreeableness were more likely to have higher level of health literacy. Agreeableness plays an important part in children’s, adolescents’, and adults’ social functioning (Kochanska and Kim, 2020). Agreeableness was positively related to parental warmth, responsiveness and authoritative parenting in general, which could promote more positive emotion regulation in children (Coplan et al., 2009). Remarkably, agreeableness was related to lower levels of stress, depression, and anxiety (Qian and Yahara, 2020; Al-Omiri et al., 2021; Nikcčvić et al., 2021). Agreeable individuals are generous, trusting and compliant, and are more likely to seek social support, and exhibit active coping and reappraisals when encountering a stressful experience (Afshar et al., 2015; Getzmann et al., 2021). Those with high levels of agreeableness were good at problem-focused coping (i.e., efforts to improve a given situation), and able to deal with the health problems resulting from negative emotion (Agbaria and Mokh, 2021). The McMaster model of family functioning, including dimensions such as problem solving, communication, roles, affective responsiveness, affective involvement and behavioral control, that has impact on the health of family members (Bello et al., 2018). Two-parent family may have sufficient family functioning which would lead to better family health status. In contrast, single-parent family may have fewer resources including time, money and social networks that might lead to poor health outcomes (Christie-Seely and Talbot, 1985; Bello et al., 2018).

The results also showed that openness was associated with health literacy. According to the definition, openness should be positively associated with searching for health-related information (Bogg and Vo, 2014). Openness broadly reflects a person’s ability to accept new experiences, both exploring internal emotions or ideas and exposing to new or unfamiliar things (Oie et al., 2020). Individuals a with high level of openness were sensitive to health information and more likely to benefit from training programs and accept health-related behaviors for health promotion (Salgado, 1997; Wagner et al., 2017). Low openness was related to personal adaptation problems and inability to understand or express one’s own feelings (Oie et al., 2020). Additionally, parents with high openness are more likely to comprehend and adjust the needs of their children in different situations (Slade, 2005). Some studies have shown that parents high in openness could sustain a satisfactory relationship and be willing to share feelings with children (Neyer and Voigt, 2004; Zhou et al., 2017).

Extraversion, as a critical predictor for parenting warmth, was labeled energetic and assertive. Extraversion had a positive correlation with physical activity, which could improve health literacy and lead to better health outcomes (Wilson et al., 2005; Krueger et al., 2009). Extroverted parents are more willing to share health information with their children, observe their children’s daily health status, and make better health decisions. Additionally, the family atmosphere could be more harmonious by exercising together. A previous study demonstrated that adolescents from a single-parent family may have remarkably higher odds of risky health behaviors (e.g., smoking and high alcohol consumption) and mental health issues (e.g., depression and suicidal ideation) (Park and Lee, 2020). Parents who scored higher on extraversion were related to positive emotional expressions interacting with children, and providing a more intimate environment and cultivating health behaviors (Prinzie et al., 2009). In contrast, negative emotions such as depression have been proven to be related to low extraversion (Chioqueta and Stiles, 2005).

Of the Big Five Personality constructs, conscientiousness reflected the extent to which a person was organized, goal directed, and followed rules (Prinzie et al., 2009; Eisenberg et al., 2014). High conscientiousness was more likely to follow social norms toward health information or health behavior, and should help family member to contribute more to the health outcome (Barrick et al., 1998; Hirsh, 2010). A previous study demonstrated that lower conscientiousness has potential explanatory relevance to risky health behavior (e.g., smoking) and childhood maltreatment (e.g., childhood neglect) (Collado et al., 2019). For example, cigarette smoking represents a risky health behavior that leads to high morbidity of lung cancer. Parents with high conscientiousness and health literacy always obey the norms and exhort their children not to smoke. However, parents may pay less attention to their children who are more likely to be exposed to the risk of poor health behavior and mental health in a single-parent family (Park and Lee, 2020).

Neuroticism is vulnerable to negative emotions such as anxiety, depression and fear (McCrae and John, 1992). Most studies demonstrated that individuals with a low level of neuroticism were more likely to have a high level of health literacy. However, our study showed that neuroticism had a positive relationship with health literacy. One possible reason for this difference could be that people with moderate neuroticism may pay more attention to health conditions, to search for health information and to use health resources rationally to maintain health and wellbeing. Neurotic people tended to have strong negative emotions to adverse events in life. Therefore, negative emotions such as anxiety and worry were inclined to cause emotional instability. Studies have shown a positive relationship between neuroticism and internalizing problems among children, adolescents, and adults, which may destroy the intimate relationship between parents and children (Malouff et al., 2005; Prinzie et al., 2009; Kotov et al., 2010). Lower neuroticism was related to more autonomy support, warmth and behavior control, which was a critical factor in maintaining positive interactions. In addition, two-parent family spent more time participating in activities with children which could improve intimate relationships (Stephan et al., 2014; Wilson and Dishman, 2015).

The present study had several limitations. First, as a cross-sectional study, it was difficult to prove causation from the findings related to health literacy and personality traits. This study found that people with high neuroticism tended to have a high level of health literacy, which was opposite to some studies. The personality score was influenced by several factors, such as age, health status, social support, and life events when they were investigated (Costa and McCrae, 1988). A longitudinal study is needed to ensure the relationship between health literacy and personality traits in two types of family structure in the future. Second, this study used a multistage random sampling method, and the number of single-parent family was much smaller than that of two-parent family. Considering the problem of ratio, two-parent family was randomly sampled at approximately 1,000, as the ratio of two-parent family to single-parent family was 2:1. Therefore, the representation of the results of two-parent families may need to be further confirmed.

## Conclusion

Overall, the results of the current study demonstrated a positively significant association between health literacy and personality traits in two types of family structure. Openness provided the strongest contribution to the related structure of canonical correlation on the personality trait set. On the other hand, healthcare and health promotion had stronger effects on the health literacy set. Hierarchical multiple regression analysis showed that education, extraversion, agreeableness, conscientiousness, neuroticism, and openness were the factors influencing health literacy in the two-parent family. Additionally, monthly income, agreeableness and openness were the factors influencing health literacy in a single-parent family. These results may offer an effective, practical and instructive approach to explaining the relation between health literacy and personality traits in two types of family structure.

## Data Availability Statement

The raw data supporting the conclusions of this article will be made available by the authors, without undue reservation.

## Ethics Statement

The studies involving human participants were reviewed and approved by the Ethics Committee of Jinan University, Guangzhou, China. Written informed consent to participate in this study was provided by the participants’ legal guardian/next of kin.

## Author Contributions

JM, WY, and SX contributed to conceiving and designing the study. ZL and LL contributed to data collection and data coding. JM analyzed the data and wrote the manuscript. All authors contributed to the article and approved the submitted version.

## Conflict of Interest

The authors declare that the research was conducted in the absence of any commercial or financial relationships that could be construed as a potential conflict of interest.

## Publisher’s Note

All claims expressed in this article are solely those of the authors and do not necessarily represent those of their affiliated organizations, or those of the publisher, the editors and the reviewers. Any product that may be evaluated in this article, or claim that may be made by its manufacturer, is not guaranteed or endorsed by the publisher.

## References

[B1] Adler N. E. Newman K. (2002). Socioeconomic disparities in health: pathways and policies. *Health Affairs* 21 60–76. 10.1377/hlthaff.21.2.60 11900187

[B2] Afshar H. Roohafza H. Keshteli A. Mazaheri M. Feizi A. Adibi P. (2015). The association of personality traits and coping styles according to stress level. *J. Res. Med. Sci.* 20 353–358. 26109990 PMC4468450

[B3] Agbaria Q. Mokh A. A. (2021). Coping with stress during the coronavirus outbreak: the contribution of big five personality traits and social support. *Int. J. Ment. Health Addict.* 1–19. [Online ahead of print]. 10.1007/s11469-021-00486-2 33500687 PMC7819145

[B4] Al-Omiri M. K. Alzoubi I. A. Al Nazeh A. A. Alomiri A. K. Maswady M. N. Lynch E. (2021). COVID-19 and personality: a cross-sectional multicenter study of the relationship between personality factors and COVID-19-Related impacts, concerns, and behaviors. *Front. Psychiatry* 12:608730. 10.3389/fpsyt.2021.608730 33716815 PMC7952985

[B5] Amato P. R. DeBoer D. D. (2001). The transmission of marital instability across generations: relationship skills or commitment to marriage? *J. Marriage Fam.* 63 1038–1051. 10.1111/j.1741-3737.2001.01038.x

[B6] Aschwanden D. Strickhouser J. E. Sesker A. A. Lee J. H. Luchetti M. Stephan Y. (2020). Psychological and behavioural responses to coronavirus disease 2019: the role of personality. *Eur. J. Pers.* 35, 51–66. 10.1002/per.2281 32836766 PMC7361622

[B7] Baker D. W. Wolf M. S. Feinglass J. Thompson J. A. Gazmararian J. A. Huang J. (2007). Health literacy and mortality among elderly persons. *Arch. Int. Med.* 167 1503–1509. 10.1001/archinte.167.14.1503 17646604

[B8] Barrick M. R. Stewart G. L. Neubert M. J. Mount M. K. (1998). Relating member ability and personality to work-team processes and team effectiveness. *J. Appl. Psychol.* 83 377–391. 10.1037/0021-9010.83.3.377

[B9] Bello C. B. Irinoye O. Akpor O. A. (2018). Health status of families: a comparative study of one-parent and two-parent families in Ondo State, Nigeria. *Afr. J. Prim. Health Care Fam. Med.* 10 e1–e8. 10.4102/phcfm.v10i1.1550 30198283 PMC6111383

[B10] Berger-Sieczkowski E. Gruber B. Stögmann E. Lehrner J. (2019). Differences regarding the five-factor personality model in patients with subjective cognitive decline and mild cognitive impairment. *Neuropsychiatrie* 33 35–45. 10.1007/s40211-018-0292-z 30328583 PMC6400874

[B11] Bloom B. Dawson D. (1991). Family structure and child health. *Am. J. Public Health* 81 1526–1528. 10.2105/ajph.81.11.1526 1951818 PMC1405685

[B12] Bogg T. Roberts B. W. (2004). Conscientiousness and health-related behaviors: a meta-analysis of the leading behavioral contributors to mortality. *Psychol. Bull.* 130 887–919. 10.1037/0033-2909.130.6.887 15535742

[B13] Bogg T. Vo P. T. (2014). Openness, neuroticism, conscientiousness, and family health and aging concerns interact in the prediction of health-related Internet searches in a representative U.S. sample. *Front. Psychol.* 5:370. 10.3389/fpsyg.2014.00370 24808880 PMC4010796

[B14] Bonaccorsi G. Lastrucci V. Vettori V. Lorini C. Florence Health Literacy Research Group. (2019). Functional health literacy in a population-based sample in Florence: a cross-sectional study using the Newest Vital Sign. *BMJ Open* 9:e026356. 10.1136/bmjopen-2018-026356 31221877 PMC6589023

[B15] Campbell S. B. (1995). Behavior problems in preschool children: a review of recent research. *J. Child Psychol. Psychiatry* 36 113–149. 10.1111/j.1469-7610.1995.tb01657.x 7714027

[B16] Carver C. S. Connor-Smith J. (2010). Personality and coping. *Annu. Rev. Psychol.* 61 679–704. 10.1146/annurev.psych.093008.100352 19572784

[B17] Chae S. (2016). Parental divorce and children’s schooling in Rural Malawi. *Demography* 53 1743–1770. 10.1007/s13524-016-0521-7 27822897 PMC5130620

[B18] Chioqueta A. P. Stiles T. C. (2005). Personality traits and the development of depression, hopelessness, and suicide ideation. *Pers. Individ. Differ.* 38 1283–1291. 10.1016/j.paid.2004.08.010

[B19] Christie-Seely J. Talbot Y. (1985). Assessing the single-parent family. *Can. Fam. Phys.* 31 1633–1639. 21274172 PMC2327857

[B20] Christopher C. Wolchik S. Tein J. Y. Carr C. Mahrer N. E. Sandler I. (2017). Long-term effects of a parenting preventive intervention on young adults’ painful feelings about divorce. *J. Fam. Psychol.* 31 799–809. 10.1037/fam0000325 28471208 PMC5662483

[B21] Collado A. Felton J. W. Taylor H. Eure A. Yi R. (2019). Conscientiousness explains the link between childhood neglect and cigarette smoking in adults from a low-income, urban area-the differential effects of sex. *Child Abuse Negl.* 88 152–158. 10.1016/j.chiabu.2018.10.015 30508683 PMC6333503

[B22] Connor-Smith J. K. Flachsbart C. (2007). Relations between personality and coping: a meta-analysis. *J. Pers. Soc. Psychol.* 93 1080–1107. 10.1037/0022-3514.93.6.1080 18072856

[B23] Coplan R. J. Reichel M. Rowan K. (2009). Exploring the associations between maternal personality, child temperament, and parenting: a focus on emotions. *Pers. Individ. Differ.* 46 241–246. 10.1016/j.paid.2008.10.011

[B24] Costa P. T. McCrae R. R. (1988). Personality in adulthood: a six-year longitudinal study of self-reports and spouse ratings on the NEO personality inventory. *J. Pers. Soc. Psychol.* 54 853–863. 10.1037/0022-3514.54.5.853 3379583

[B25] DeWalt D. A. Berkman N. D. Sheridan S. Lohr K. N. Pignone M. P. (2004). Literacy and health outcomes. *J. Gen. Int. Med.* 19 1228–1239. 10.1111/j.1525-1497.2004.40153.x 15610334 PMC1492599

[B26] Duong T. V. Aringazina A. Kayupova G. Nurjanah, Pham T. V. Pham K. M. (2019). Development and validation of a new short-form health literacy instrument (HLS-SF12) for the general public in six Asian Countries. *Health Lit. Res. Pract.* 3 e91–e102. 10.3928/24748307-20190225-01 31294310 PMC6607763

[B27] Eisenberg N. Duckworth A. L. Spinrad T. L. Valiente C. (2014). Conscientiousness: origins in Childhood? *Dev. Psychol.* 50 1331–1349. 10.1037/a0030977 23244405 PMC3610789

[B28] Getzmann S. Digutsch J. Kleinsorge T. (2021). COVID-19 pandemic and personality: agreeable people are more stressed by the feeling of missing. *Int. J. Environ. Res. Public Health* 18:10759. 10.3390/ijerph182010759 34682500 PMC8535900

[B29] Harsch S. Jawid A. Jawid E. Saboga-Nunes L. Sorensen K. Sahrai D. (2021). Health literacy and health behavior among Women in Ghazni, Afghanistan. *Front. Public Health* 9:629334. 10.3389/fpubh.2021.629334 33748067 PMC7969710

[B30] Herke M. Knochelmann A. Richter M. (2020). Health and well-being of adolescents in different family structures in germany and the importance of family climate. *Int. J. Environ. Res. Public Health* 17:6470. 10.3390/ijerph17186470 32899489 PMC7559242

[B31] Hirsh J. B. (2010). Personality and environmental concern. *J. Environ. Psychol.* 30 245–248. 10.1016/j.jenvp.2010.01.004

[B32] Huang I. C. Lee J. L. Ketheeswaran P. Jones C. M. Revicki D. A. Wu A. W. (2017). Does personality affect health-related quality of life? A systematic review. *PLoS One* 12:e0173806. 10.1371/journal.pone.0173806 28355244 PMC5371329

[B33] Ishikawa H. Yamaguchi I. Nutbeam D. Kato M. Okuhara T. Okada M. (2018). Improving health literacy in a Japanese community population—A pilot study to develop an educational programme. *Health Expectations Int. J. Public Participat. Health Care Health Pol.* 21 814–821. 10.1111/hex.12678 29602238 PMC6117484

[B34] Iwasa H. Yoshida Y. (2020). Personality and health literacy among community-dwelling older adults living in Japan. *Psychogeriatrics* 20 824–832. 10.1111/psyg.12600 32812314

[B35] Kochanska G. Kim S. (2020). Children’s early difficulty and agreeableness in adolescence: testing a developmental model of interplay of parent and child effects. *Dev. Psychol.* 56 1556–1564. 10.1037/dev0001023 32510231 PMC8262374

[B36] Kotov R. Gamez W. Schmidt F. Watson D. (2010). Linking “Big” personality traits to anxiety, depressive, and substance use disorders: a meta-analysis. *Psychol. Bull.* 136 768–821. 10.1037/a0020327 20804236

[B37] Krueger K. R. Wilson R. S. Kamenetsky J. M. Barnes L. L. Bienias J. L. Bennett D. A. (2009). Social engagement and cognitive function in old age. *Exp. Aging Res.* 35 45–60. 10.1080/03610730802545028 19173101 PMC2758920

[B38] Lastrucci V. Lorini C. Caini S. Bonaccorsi G. Alti E. Baglioni S. (2019). Health literacy as a mediator of the relationship between socioeconomic status and health: a cross-sectional study in a population-based sample in Florence. *PLoS One* 14:e0227007. 10.1371/journal.pone.0227007 31869381 PMC6927637

[B39] Laursen B. P. Hafen C. A. Rubin K. H. Booth-LaForce C. Rose-Krasnor L. (2010). The distinctive difficulties of disagreeable youth. *Merrill-Palmer Q.* 56 80–103. 10.1353/mpq.0.0040 35812133 PMC9269991

[B40] Malouff J. M. Thorsteinsson E. B. Schutte N. S. (2005). The relationship between the five-factor model of personality and symptoms of clinical disorders: a meta-analysis. *J. Psychopathol. Behav. Assess.* 27 101–114. 10.1007/s10862-005-5384-y

[B41] Manganello J. A. (2007). Health literacy and adolescents: a framework and agenda for future research. *Health Educ. Res.* 23 840–847. 10.1093/her/cym069 18024979

[B42] Martin L. T. Chen P. (2015). “Child health and school readiness: the significance of health literacy,” in *Health and Education in Early Childhood*, eds Reynolds A. J. Rolnick A. J. Temple J. A. (Cambridge: Cambridge University Press), 346–368. 10.1017/cbo9781139814805.018

[B43] McCrae R. R. John O. P. (1992). An introduction to the five-factor model and its applications. *J. Pers.* 60 175–215. 10.1111/j.1467-6494.1992.tb00970.x 1635039

[B44] Neyer F. J. Voigt D. (2004). Personality and social network effects on romantic relationships: a dyadic approach. *Eur. J. Pers.* 18 279–299. 10.1002/per.519

[B45] Nikcčvić A. V. Marino C. Kolubinski D. C. Leach D. Spada M. M. (2021). Modelling the contribution of the Big Five personality traits, health anxiety, and COVID-19 psychological distress to generalised anxiety and depressive symptoms during the COVID-19 pandemic. *J. Affect. Disord.* 279 578–584. 10.1016/j.jad.2020.10.053 33152562 PMC7598311

[B46] Kim S. Kochanska G. (2019). Evidence for childhood origins of conscientiousness: testing a developmental path from toddler age to adolescence. *Dev. Psychol.* 55 196–206. 10.1037/dev0000608 30382718 PMC6296862

[B47] National Health Commission of the People’s Republic of China. (2021). *The Health Literacy Level of Chinese Residents Rose to 23.15% in 2020.* Available online at: http://www.nhc.gov.cn/xcs/s7847/202104/6cede3c9306a41eeb522f076c82b2d94.shtml (accessed April 1, 2021).

[B48] Norman W. T. (1963). Toward an adequate taxonomy of personality attributes: replicated factor structure in peer nomination personality ratings. *J. Abnorm. Soc. Psychol.* 66 574–583. 10.1037/h0040291 13938947

[B49] Nutbeam D. (2000). Health literacy as a public health goal: a challenge for contemporary health education and communication strategies into the 21st century. *Health Promot. Int.* 15 259–267. 10.1093/heapro/15.3.259

[B50] Oie M. G. Aarnes I. E. Eilertsen L. H. Soderstrom K. Ystrom E. Hakansson U. (2020). High levels of the openness trait are associated with better parental reflective functioning in mothers with substance use disorders. *Addict. Behav. Rep.* 12:100318. 10.1016/j.abrep.2020.100318 33364326 PMC7752705

[B51] Park H. Lee K. S. (2020). The association of family structure with health behavior, mental health, and perceived academic achievement among adolescents: a 2018 Korean nationally representative survey. *BMC Public Health* 20:510. 10.1186/s12889-020-08655-z 32299419 PMC7164151

[B52] Prinzie P. Stams G. J. Dekovic M. Reijntjes A. H. Belsky J. (2009). The relations between parents’ Big Five personality factors and parenting: a meta-analytic review. *J. Pers. Soc. Psychol.* 97 351–362. 10.1037/a0015823 19634980

[B53] Qian K. Yahara T. (2020). Mentality and behavior in COVID-19 emergency status in Japan: influence of personality, morality and ideology. *PLoS One* 15:e0235883. 10.1371/journal.pone.0235883 32649687 PMC7351180

[B54] Rammstedt B. John O. P. (2007). Measuring personality in one minute or less: a 10-item short version of the Big Five Inventory in English and German. *J. Res. Pers.* 41 203–212. 10.1016/j.jrp.2006.02.001

[B55] Rask M. Uusiautti S. Määttä K. (2014). The fourth level of health literacy. *Int. Q. Commun. Health Educ.* 34 51–71. 10.2190/IQ.34.1.e 24366022

[B56] Roberts B. W. Jackson J. J. Burger J. Trautwein U. (2009). Conscientiousness and externalizing psychopathology: overlap, developmental patterns, and etiology of two related constructs. *Dev. Psychopathol.* 21 871–888. 10.1017/S0954579409000479 19583888

[B57] Salgado J. F. (1997). The five factor model of personality and job performance in the European Community. *J. Appl. Psychol.* 82 30–42. 10.1037//0021-9010.82.1.30 9119797

[B58] Segerstrom S. C. (2020). Personality and incident Alzheimer’s disease: theory, evidence, and future directions. *J. Gerontol B Psychol. Sci. Soc. Sci.* 75 513–521. 10.1093/geronb/gby063 29846724 PMC7768711

[B59] Sentell T. Vamos S. Okan O. (2020). Interdisciplinary perspectives on health literacy research around the world: more important than ever in a time of COVID-19. *Int. J. Environ. Res. Public Health* 17:3010. 10.3390/ijerph17093010 32357457 PMC7246523

[B60] Slade A. (2005). Parental reflective functioning: an introduction. *Attach. Hum. Dev.* 7 269–281. 10.1080/14616730500245906 16210239

[B61] Sorensen K. Van den Broucke S. Fullam J. Doyle G. Pelikan J. Slonska Z. (2012). Health literacy and public health: a systematic review and integration of definitions and models. *BMC Public Health* 12:80. 10.1186/1471-2458-12-80 22276600 PMC3292515

[B62] Soutter A. R. B. Bates T. C. Mottus R. (2020). Big five and HEXACO personality traits, proenvironmental attitudes, and behaviors: a meta-analysis. *Perspect. Psychol. Sci.* 15 913–941. 10.1177/1745691620903019 32384257 PMC7333518

[B63] Stephan Y. Boiché J. Canada B. Terracciano A. (2014). Association of personality with physical, social, and mental activities across the lifespan: findings from US and French samples. *Br. J. Psychol.* 105 564–580. 10.1111/bjop.12056 24182200

[B64] van Scheppingen M. A. Chopik W. J. Bleidorn W. Denissen J. J. A. (2019). Longitudinal actor, partner, and similarity effects of personality on well-being. *J. Pers. Soc. Psychol.* 117 e51–e70. 10.1037/pspp0000211 30102060 PMC6374220

[B65] Wagner M. T. Mithoefer M. C. Mithoefer A. T. MacAulay R. K. Jerome L. Yazar-Klosinski B. (2017). Therapeutic effect of increased openness: investigating mechanism of action in MDMA-assisted psychotherapy. *J. Psychopharmacol. (Oxford)* 31 967–974. 10.1177/0269881117711712 28635375 PMC5544120

[B66] Wharf Higgins J. Begoray D. MacDonald M. (2009). A social ecological conceptual framework for understanding adolescent health literacy in the health education classroom. *Am. J. Commun. Psychol.* 44 350–362. 10.1007/s10464-009-9270-8 19838790

[B67] Wilson K. E. Dishman R. K. (2015). Personality and physical activity: a systematic review and meta-analysis. *Pers. Individ. Differ.* 72 230–242. 10.1016/j.paid.2014.08.023

[B68] Wilson R. S. Krueger K. R. Gu L. Bienias J. L. Mendes De Leon C. F. Evans D. A. (2005). Neuroticism, extraversion, and mortality in a defined population of older persons. *Psychosomatic Med.* 67 841–845. 10.1097/01.psy.0000190615.20656.83 16314587

[B69] Yong-bao W. Jing X. (2019). An empirical study on the relationship between personality traits and subjective well-being of students from single-parent family. *J. Liaoning Higher Vocat.* 21 104–108.

[B70] Zarcadoolas C. Pleasant A. Greer D. S. (2005). Understanding health literacy: an expanded model. *Health Promot. Int.* 20 195–203. 10.1093/heapro/dah609 15788526

[B71] Zhou Y. Wang K. Chen S. Zhang J. Zhou M. (2017). An exploratory investigation of the role of openness in relationship quality among emerging adult Chinese couples. *Front. Psychol.* 8:382. 10.3389/fpsyg.2017.00382 28360875 PMC5350098

